# Supplementary material for: A Universal Biofilm Reactor Sensor for the Determination of Biochemical Oxygen Demand of Different Water Areas
Source: Molecules. 2022 Aug 8;27(15):5046. doi: 10.3390/molecules27155046 (PMC9370119; doi:10.3390/molecules27155046)
Supplement: Supplementary file 1 [file molecules-27-05046-s001.zip › molecules-1815792-supplementary.pdf]

Supporting Information

A universal biofilm reactor sensor for the determination of biochemical oxygen demand of different water areas

Liang Wang, Huan Lv, Qian Yang, Yiliang Chen, Junjie Wei, Yiyuan Chen, Ci'en Peng, Changyu Liu, Xiaolong Xu *, Jianbo Jia *

School of Biotechnology and Health Sciences, Wuyi University, Jiangmen 529020, China

Table S1. List of Experimental Reagents

NO.	Reagent	formula	factory
1	Glucose	$C_6H_{12}O_6$	Macklin
2	Glutamate	$C_5H_9NO_4$	Macklin
3	Sodium chloride	NaCl	Guangzhou Chemical Reagent
4	Magnesium chloride hexahydrate	$MgCl_2 \cdot 6H_2O$	Guangzhou Chemical Reagent
5	Anhydrous sodium sulfate	Na_2SO_4	Guangzhou Chemical Reagent
6	Anhydrous calcium chloride	$CaCl_2$	Guangzhou Chemical Reagent
7	Potassium chloride	KCl	Guangzhou Chemical Reagent
8	Sodium bicarbonate	$NaHCO_3$	Guangzhou Chemical Reagent
9	Sodium bromide	NaBr	Guangzhou Chemical Reagent
10	Leucine	$C_6H_{13}NO_2$	Macklin
11	Lysine	$C_6H_{14}N_2O_2$	Macklin
12	Malic acid	$C_4H_6O_5$	Macklin
13	Sorbitol	$C_6H_{14}O_6$	Macklin
14	N-butanol	$C_4H_{10}O$	Guangzhou Chemical Reagent
15	Fumaric acid	$C_4H_4O_4$	Macklin
16	Ethyl acetate	$C_4H_8O_2$	Guangzhou Chemical Reagent
17	Xylose	$C_5H_{10}O_5$	Macklin

18	Galactose	$C_6H_{12}O_6$	Macklin
19	Ferric chloride	$FeCl_3$	Guangzhou Chemical Reagent
20	Magnesium sulphate	$MgSO_4$	Guangzhou Chemical Reagent
21	Potassium dihydrogen Phosphate	KH_2PO_4	Guangzhou Chemical Reagent
22	Disodium hydrogen phosphate	Na_2HPO_4	Guangzhou Chemical Reagent
23	Ammonium chloride	NH_4Cl	Guangzhou Chemical Reagent
